# Aging of Vascular System Is a Complex Process: The Cornerstone Mechanisms

**DOI:** 10.3390/ijms23136926

**Published:** 2022-06-22

**Authors:** Anastasia V. Poznyak, Nikolay K. Sadykhov, Andrey G. Kartuesov, Evgeny E. Borisov, Vasily N. Sukhorukov, Alexander N. Orekhov

**Affiliations:** 1 Institute for Atherosclerosis Research, Osennyaya 4-1-207, 121609 Moscow, Russia; vnsukhorukov@gmail.com; 2 Laboratory of Angiopathology, Institute of General Pathology and Pathophysiology, 8 Baltiiskaya Street, 125315 Moscow, Russia; drawnman@mail.ru (N.K.S.); kartuesv@gmail.com (A.G.K.); 3 Laboratory of Cellular and Molecular Pathology of Cardiovascular System, Petrovsky National Research Centre of Surgery, 2, Abrikosovsky Lane, 119991 Moscow, Russia; borisovevgenij5@gmail.com

**Keywords:** aging, cardiovascular system, arteries, CVD

## Abstract

Aging is one of the most intriguing processes of human ontogenesis. It is associated with the development of a wide variety of diseases affecting all organs and their systems. The victory over aging is the most desired goal of scientists; however, it is hardly achievable in the foreseeable future due to the complexity and ambiguity of the process itself. All body systems age, lose their performance, and structural disorders accumulate. The cardiovascular system is no exception. And it is cardiovascular diseases that occupy a leading position as a cause of death, especially among the elderly. The aging of the cardiovascular system is well described from a mechanical point of view. Moreover, it is known that at the cellular level, a huge number of mechanisms are involved in this process, from mitochondrial dysfunction to inflammation. It is on these mechanisms, as well as the potential for taking control of the aging of the cardiovascular system, that we focused on in this review.

## 1. Introduction

Changes in arterial structure and function accompanying aging lead to an increased risk of cardiovascular diseases (CVD). Thus, understanding the mechanisms by which age affects the vascular system can help to avoid altogether or to reduce the high risk of developing cardiovascular diseases in elderly people [1].

Several new (preliminary) clinical studies have found that the most important vascular changes occur with aging and described 2 key traits: (1) generalized endothelial dysfunction and (2) stiffness of the central artery. As for generalized endothelial dysfunction, vascular aging alters the endothelium function and the cells that cover the lumen of blood vessels [2]. Endothelial dysfunction includes a decrease in vasodilatory and antithrombotic properties, with an elevation in oxidative stress and inflammatory cytokines, which favor atherogenesis and thrombosis and predispose to cardiovascular diseases. Both human and experimental studies have proven a reduction in the bioavailability of nitric oxide (NO), a major mediator of vasorelaxation and antiatherogenic processes that are the foundation of age-related endothelial dysfunction [3].

With aging, the elasticity of arteries, especially the aorta, decreases. This leads to arterial stiffness, which is, at least in part, the result of gradual fragmentation and loss of elastin fibers and accumulation of stiffer collagen fibers. The risk of hypertension and the range of various disorders are tightly linked to increased arterial stiffness [4]. Vascular calcification is specific for aging and vascular stiffness. The development of calcification is accelerated in patients with hypertension, diabetes mellitus and other disorders. However, an exact mechanism linking calcification with aging is still unclear [5].

A decrease in the bioavailability of NO may occur due to a reduction in synthesis or high degradation of NO. Under standard circumstances, endothelial nitric oxide synthase (eNOS) produces NO from l-arginine in the presence of the cofactor tetrahydrobiopterin (BH4) [6]. Even though the studies have differences in the expression of the eNOS protein with age, the latest data report an age-related change in eNOS function, called eNOS uncoupling. This effect is at least partially associated with a reduction in the availability of BH4, which leads to an inhibition of the release of NO and an elevation in the production of the highly oxidative superoxide anion (O_2_^−^) [7,8]. In addition, the availability of the NOS substrate, l-arginine, may also limit the production of NO. As a result, the activity of arginase, an enzyme that competes with eNOS for l-arginine, rises with age, giving a plausible mechanism for reducing the synthesis of NO. However, most of this mechanical evidence has been discovered through animal studies, which indicates the necessity for further human studies regarding the age restriction of NO synthesis [9]. 

In addition, the aging process is able to elevate the degradation of NO due to excessive concentrations of reactive oxygen species (ROS), partially mediated by chronic inflammation, which leads to a vicious circle that drains NO [10]. For instance, an age-dependent elevation in vascular and blood tumor necrosis factor (TNF)-α is associated with high expression of nicotinamide adenine dinucleotide phosphate (NADPH) oxidase. The increased activity of NADPH oxidase results in a secondary production of O_2_^−^, which, for its part, reacts with NO to form peroxynitrite (ONOO^−^), a powerful oxidant involved in the nitrosylation of antioxidant enzymes and eNOS. In addition to inflammatory cytokines, the renin-angiotensin-aldosterone system (RAAS) is able to promote an age-related elevation in NO inactivation [11]. With age, the activity of RAAS and the concentration of angiotensin II, the main effector of RAAS, increases, which leads to a rise in the production of ROS due to the activation of NADPH oxidase. An age-related elevation in ROS, in turn, is able to stimulate vascular inflammation. Hydrogen peroxide (H_2_O_2_) activates the nuclear factor kappa-B (NF-κB), which enhances the transcription of pro-inflammatory genes, resulting in elevated expression of TNF-α, interleukin 6, chemokines, and adhesion molecules which participate in atherogenesis [12].

## 2. Main Mechanisms Involved in Vascular Aging

**Figure 1:**
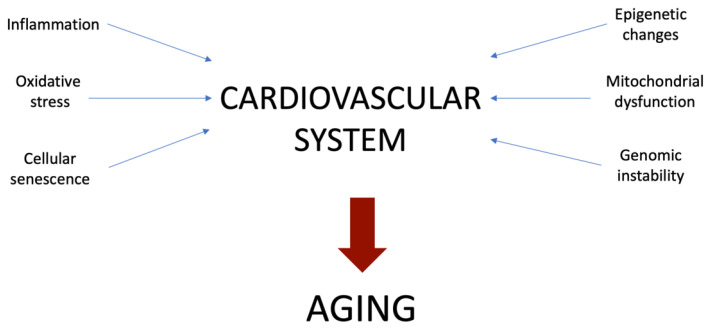
Schematic representation of main cellular mechanisms contributing to aging.

As was postulated previously, aging is a complex process involving various mechanisms and their interplay (see Figure 1). Here we summarize the main impact of the most essential cellular processes that contribute to aging.

## 3. Inflammation

During the process of aging, a shift towards the pro-inflammatory phenotype with elevated expression of inflammatory cytokines, adhesion molecules, and chemokines from ECs has been observed. These pro-inflammatory cytokines include interleukin (IL)-6, IL-1β, cellular adhesion molecules, tumor necrosis factor-alpha (TNF-α), and monocyte chemoattractant protein-1 [13]. The proinflammatory response is associated with the activation of nuclear factor-kappaB (NF-KB), signaling that the key factor of nuclear transcription favors the expression of inflammatory cytokines in endothelial dysfunction and cardiovascular diseases. A new molecular marker of vascular inflammation is the NACHT, LR, and PYD inflammasome, which contains protein 3 (NLRP3). Activated NLRP3 undergoes oligomerization and stimulates recruitment of procaspase-1, and activation of caspase-1 is an amplifier of a variety of proinflammatory pathways, including NF-KB, chemokines, and ROS [14]. At the same time, the elevated transmission of NF-KB signals leads to favorable feedback, which, in turn, additionally stimulates the assembly of the NLRP3 inflammasome, processing, and maturation of pro-IL-1. Due to changes in the brain structure in elderly people, circulating biomarkers of inflammation, such as C-reactive protein, IL-6, are elevated. Prolonged exposure to TNF-α leads to early aging of the EC, which can be avoided by suppressing the activation of NF-KB. This fact gives rise to the hypothesis that inflammation leads to premature aging of the EC. Thus, human aging is a chronic, systemic and low-grade pro-inflammatory condition, and this phenomenon has been defined as “inflammaging” [15].

## 4. Oxidative Stress

It is well known that oxidative stress is essential in both age-related EC dysfunction and arterial dysfunction. There are many sources of ROS in cells, such as hydrogen peroxide, peroxynitrite, and hydroxyl radical. ROS are formed not only as a result of mitochondrial respiration, but also as a result of peroxisomal β-oxidation of free fatty acids, xanthine oxidase, lipoxygenase, nicotinamide adenine dinucleotide phosphate (NADPH) oxidase (NOX), microsomal enzymes P-450, cyclooxygenases, and prooxidant heme molecules [16].

In addition, the uncoupling of endothelial NO synthase (ENOS) also favors the development of peroxynitrite. Even though physiological levels of ROS are required to maintain normal cellular function, ROS overproduction leads to extremely undesirable effects, such as, for example, changes in DNA transcription, interruption of numerous redox-sensitive signaling pathways, disruption of cellular structure and function, inflammation, as well as dysfunction of entire organs [17].

NOX is one of the key enzymes responsible for most of the formation of ROS in cardiovascular diseases. It was found that isoforms NOS 1, NX2, NX4, and NOX 5 stimulate EC dysfunction, inflammation, and apoptosis in diseases such as atherosclerosis, hypertension, and diabetes. In patients with the acute coronary syndrome, microparticles of endothelial origin led to early aging of the EC through NOX-mediated activation of mitogen-activated protein kinases and phosphoinositide-3-kinase/protein kinase B. Oxidative stress-stimulated cellular stress and trauma are the driving factors leading to early aging of the EC, vascular stiffness and the development of age-related cardiovascular diseases [18].

## 5. Mitochondrial Dysfunction

As new data have shown that mitochondria function in networks, mitochondrial research has undergone a paradigm shift. Consequently, mitochondria are no longer regarded as static isolated organelles intended only for the production of ATP in the cell. They are more like extremely mobile objects forming dynamic networks of long tubules [19,20,21]. Both functional and morphological behavior of mitochondria in cells has a rather large impact on the bioenergetic status of cells, tissues, and organs and adds features that are characteristic of complex systems: reliability, redundancy of functions, and plasticity. These characteristics give the system adaptive flexibility, which is essential for adapting to changing stresses and metabolic requirements [22].

The morphology and functions of mitochondrial networks are controlled by uninterrupted fusion and division cycles, which play a key role in determining the shape of organelles and in transmitting redox-sensitive signals, redistributing metabolites and proteins, keeping the wholeness of mtDNA, implementing metabolic processes and regulating QC and cell death pathways [23]. For example, the functionality of defective mitochondria is able to be supplemented and theoretically recreated by connecting them with neighboring intact mitochondria. If severely damaged, mitochondria are separated from the mitochondrial network by division and, as a result, are removed by mitophagy. Consequently, the dynamics of mitochondria and autophagy form the QC axis; dysfunction is considered to favor the aging of the cardiovascular (CV) system and cardiovascular diseases [24].

The balance between unification and separation depends on the complex mechanism of mitochondrial dynamics. Among the most detailed described factors of mitochondrial fusion in mammals are the dynamin-related GTPases mitofusin 1 and 2 (Mfn1 and Mfn2), which are responsible for the binding and fusion of external mitochondrial membranes between 2 organelles, and optical atrophy protein 1 (OPA1), which regulates the fusion of internal membranes. Mitochondrial division is controlled by dynamin-bound protein 1 (Drp1) and division protein 1 (Fis1) [25].

In recent times, the function of mitochondrial dynamics in the CV system has been studied, for example, in primary vascular endothelial cells, vascular smooth muscle cells (VSMCS), cardiac cell lines, and neonatal cardiomyocytes, in which mitochondria are located in a filamentous network and continuously undergo fusion and division processes [26]. In mature cardiomyocytes, mitochondria, at least IFMS, are organized into a more regular crystal lattice. It remains unclear whether this spatial distribution can interfere with the processes of fusion-division. In any case, recent data show that the dynamics of mitochondria are related to the physiology of the mature human heart, despite the fact that mitochondria are located in a special way in this organ [27].

Changes in the dynamics of mitochondria contribute to several cardiac and vascular events, including VSMC proliferation, cardiac development, and differentiation, stem cell differentiation, cardiomyocyte hypertrophy, myocardial damage in ischemia-reperfusion (I/R), and CHF. For example, excessive cleavage (and/or lowered fusion) might be harmful in ischemia-reperfusion injury, diabetes, hyperglycemia, and heart failure [28,29,30]. It has been shown that under I/R conditions, suppression of mitochondrial division is cardioprotective. Some recent studies also demonstrated that in order to maintain normal mitochondrial function and avoid the development of cardiac hypertrophy and heart failure, fused proteins OPA1, Mfn1 and Mfn2 are required [31].

Interestingly, some of the proteins which are forming mitochondria have pleiotropic effects that do not depend on their feature to modulate the morphology of mitochondria. Paradigmatic in this context is Mfn2; it mediates fusion and participates in apoptosis (interacting with Bak and Bax), mitophagy (as a substrate of the mitophagy-related Parkin protein), and in binding between mitochondria and the endoplasmic reticulum [32]. It is noteworthy that Mfn2 suppresses the proliferation of VSMC in various vascular proliferative conditions and activates VSMC apoptosis mediated by oxidative stress [33].

In aged and postmitotic endothelial human umbilical vein endothelial cells (HUVECs), a well-proven cell culture model for tracking mitochondrial activity and dysfunction during vascular aging, mitochondria show obvious morphological changes, loss of Δ*ψ*_m_, and depletion of mtDNA. Fusion and fission activity in aged HUVECs is lower, suggesting that these processes are variable during aging, which may further lead to the storing of damaged mitochondria [34,35].

Recent studies shows that mitochondrial dynamics can be modulated by various compounds targeting the fusion or fission proteins. For example, the treatment with Mdivi-1, inhibitor of DRP1, is able to significantly decrease atherosclerotic lesion formation in streptozotocin-induced diabetic ApoE−/− mice [36]. This compound appeared to inhibit the proliferation and migration of VSMC via the attenuation of ROS production and DRP1 phosphorylation [37]. Ilexgenin A, which is a novel pentacyclic triterpenoid, was shown to decrease atherosclerosis in ApoE−/− mice. This compound inhibits mitochondrial fission and stimulates DRP1 degradation dependent on Nrf2-induced proteasome subunit beta 5 in ECs. This, in turn, contributes to mitochondrial fission repression, relieving endothelial dysfunction [38].

Despite the preliminary nature of the existing data, these exact data prove that optimization of mitochondrial dynamics can be used as a therapeutic agent in the fight against both CV aging and CVD. In order to understand the significance of mitochondrial division and fusion in the CV system, both in health and in the presence of diseases, additional research is necessary [39].

## 6. Cellular Senescence

The storage of a large number of aging cells in the vessel wall and the heart can lead to both structural and functional deterioration of the CV system as we get older. There is important evidence of telomere shortening during cell aging [40]. Telomeres consist of repeating sequences of nucleotides (TTAGGG) at the ends of mammalian chromosomes, which maintain the stability and integrity of chromosomes, without giving a chance for destruction or fusion with neighboring chromosomes [41].

With each cell division, the telomeric DNA is shortened; and this happens until the cells being at a critical length finally lose the function of capping at the ends of the chromosomes, activating the control points of DNA damage, cell aging, and apoptosis [42]. In the presence of cardiovascular diseases, telomere shortening is of particular importance. Leukocyte telomere length (LTL) is mostly associated with vascular cell aging, aortic valve stenosis, risk factors for cardiovascular diseases (i.e., hypertension, type 2 diabetes, obesity, and smoking), and the risk of atherothrombotic events [43]. Nevertheless, the causal nature of these relationships is not yet fully clear. People with clinical and subclinical signs of atherosclerosis showed a drop in LTL in contrast to healthy control groups, even after adjusting for characteristics such as age, gender, and race [44]. 

In a case-control study, it was observed that patients with shorter LTL (only middle-age and older individuals were included) had an increased presence of ischemic (OR: 1.37; 95% confidence interval [CI]: 1.06–1.77) and hemorrhagic stroke (OR: 1.48; 95% CI: 1.08–2.02) compared with the highest telomere length tercile [45]. At the same time, patients with low LTL had a much higher risk of plaque occurrence (risk ratio: 1.49; 95% CI: 1.09–2.03), as well as a higher risk of their plaque progression (risk ratio: 1.61; 95% CI: 1.26–2.07). A recent meta-analysis, which also included prospective and retrospective studies of the relationship between LTL and CHD (43,725 people participated in the study, of whom 8400 had CVD), demonstrated that patients with the shortest LTL had a higher relative risk (RR) for CHD (RR: 1.54; 95% CI: 1.30–1.83) and cerebrovascular diseases (HR: 1.42; 95% CI: 1.11–1.81) [46].

It is important to note that aging is not the only factor affecting the length of telomeres. Thus, an increased oxidative stress levels stimulate telomere shortening. Such factors as obesity, gender, and smoking can also contribute to shortening of telomeres [47]. 

Regarding these findings, it seems to be important to include both LTL and aging in cardiovascular risk assessment [48].

Age-related defects of adrenergic signaling and calcium processing are crucial aspects in the context of cellular aging. As we age, plasma levels of norepinephrine elevate significantly due to a reduction in plasma clearance and elevated spillover from the tissues [49]. A decrease in the catecholamine reuptake transporter localized in the sympathetic nerve endings also favors an elevation in the concentration of catecholamines with age. In total, all changes steadily decrease adrenergic sensitivity, which results in beta-adrenergic desensitization [50].

As a result, this phenomenon leads to a reduction in the number, affinity, and internalization of β-adrenergic receptors (namely, the subtype of β1-adrenergic receptors) in combination with a decrease in membrane adenylate cyclase activity or cellular production of cyclic adenosine monophosphate [51]. These defects of autonomic modulation favor chronotropic incompetence and a decrease in the LV inotropic reserve, which affects exercise tolerance [52].

Calcium reuptake decreased by the sarcoplasmic myocardial reticulum by calcium adenosine triphosphatase (SERCA2a) is another important feature of cardiomyocyte aging, leading to disruption of early LV diastolic filling and compensatory elevation in atrial contraction. The amplitude of the calcium transition process decreases with age, becoming 3.2 times smaller in myocytes in patients aged ≥ 75 years compared with patients younger than 55 years [53]. In addition, there may be an age delay in the propagation of the calcium transistor from the sarcolemma to the center of the cell. With age, myocytes decrease SERCA2 expression by restricting the amount of calcium released by the sarcoplasmic reticulum, as well as inactivation of calcium channels by I_Ca_ stimulated by calcium release [46]. These changes reduce mechanical efficiency and electrophysiological properties, as well as elevate the risk of arrhythmias (i.e., atrial fibrillation) in elderly people [54].

## 7. Genomic Instability

Since the appearance of the first formulations of the Theory of Somatic Mutations of aging, a lot of information has been accumulated pro et contra the causal role of DNA damage and the accumulation of mutations in aging [55]. There is evidence that old cells have the ability to store various types of genetic damage, including somatic mutations, chromosomal aneuploidy, copy number variations, and telomere shortening. Nevertheless, despite the results achieved, further research will be required to study the role of genomic instability in vascular aging [56].

Hypotheses that predict that genome instability has a certain significance in vascular aging tend to focus on the main role of DNA damage caused by oxidative stress, showing how these hypotheses and the hypothesis of aging associated with oxidative stress are interrelated [57]. It is important to note that, compared to many other cell types, endothelial cells appear to have less efficient DNA repair pathways. It is noteworthy that popular types of interventions that cause extensive DNA damage (e.g., irradiation of the entire brain) result in notable phenotypic and functional changes in endothelial cells, favoring microvascular dilution, vascular dilatation, and pro-inflammatory changes, simulating several aspects of the aging phenotype [58].

In order to prevent the spread of damaged DNA, replicative aging is triggered in vascular endothelial cells. Within the framework of recent events, there is an assumption that this might be a key mechanism due to which DNA damage provokes vascular aging [59].

In order to determine the causal role of DNA damage in vascular aging, one laboratory study showed that mouse models with genomic instability, caused by defective nucleotide excision repair genes (ERCC1, XPD), show vascular phenotypes similar to aging, including endothelial dysfunction, elevated vascular stiffness, elevated presence of aging cells and hypertension [60]. Nevertheless, these mouse models also showed serious diseases of the liver, kidneys, bone marrow, neurological, and/or bone phenotypes that are associated with a life expectancy reduction. It is also not entirely clear how closely these phenotypes truly mimic real aging [61,62,63]. 

It has also been demonstrated that mice with a genetic deficiency of the spindle assembly checkpoint protein BubR1, which favors progressive aneuploidy, also show vascular phenotypes similar to aging, including endothelial dysfunction, elevated vascular stiffness, media atrophy, and fibrosis. However, this mouse model also illustrates the short lifespan that is linked with serious functional deficiencies in many organs, including cachectic dwarfism and lordokyphosis. Therefore, it is still not clear how relevant this model is for normal aging [64]. It has also been hypothesized that there is a connection between single-nucleotide polymorphisms of human DNA repair genes and vascular stiffness, but at this stage, the mechanistic role of DNA repair pathways in the genesis of age-related vascular diseases in humans has yet to be determined [65].

Some studies have shown that elevated oxidative DNA damage and elevated expression of multiple biomarkers of DNA double-strand ruptures were detected in atherosclerotic plaques. In transgenic mouse models, acceleration or, conversely, deceleration of the recovery of double-strand ruptures, on the one hand, have less effect on atherogenesis, and on the other hand, significantly change the stability of plaques [66].

It has been found that accelerated vascular pathologies can occur among children with Hutchinson–Gilford progeria syndrome and other laminopathies, which ultimately result in fatal MI or stroke at a very young age [67]. There is data confirming that the DNA damage reaction caused by genetic dysfunction of the nuclear plate results in phenotypic changes, such as aging in VSMC. However, we have yet to show that these pathways also contribute to “normal” vascular aging [68].

It is believed that the violation of the mechanisms that are responsible for maintaining the appropriate length and functionality of telomeres has a certain significance in vascular aging and hypertension by inducing cellular senescence [69].

## 8. Epigenetic Changes

The most studied and prevalent modification of DNA is methylation of cytosine (5-methylcytosine or 5mC). The status of 5mC groups of CpGs is related to the onset of disease and mortality, and thus serves as the basis for several pan-tissue clocks which were developed to assess “biological age” [70]. For instance, two epigenetic clocks Grim Age and PhenoAge, which were trained in chronological age and biomarkers based on blood, relate to the time when cardiovascular diseases occur. Despite the fact that the specific mechanistic basis for these clocks is still not fully understood, the key affected areas of the genome, apparently, are polycomb targets and those that are directly next to the developmental genes [71]. Accordingly, DNA methylase profiling in purified cardiomyocytes of mice that had suffered heart failure showed methylation patterns that were similar to those in newborns. Also, there is an independent study of epigenome-wide associations, which investigated the relationship between DNA methylation and cases of cardiovascular diseases, and revealed two CpG modules in human cohorts: the first was linked with developmental genes and the second one with immune functions [72].

Regarding the activation of developmental genes in diseased hearts, the landscape of 5-hydroxymethylcytosine (5hmC, the oxidative product of 5mC) in cardiomyocytes that were obtained from developing and hypertrophied hearts partially resemble the neonate-like signature [73]. It was demonstrated that 5hmC, which correlates unfavorably with gene transcription, was decreased in contrast to mitochondrial genes and elevated in contrast to enhancers and gene bodies of fetal genes, such as Myh7, thereby activating them repeatedly [74]. 

The study of DNA methylation in healthy and atherosclerotic lesions from aortic samples selected by a donor showed focal hypermethylation in the affected tissue in repetitive and non-repeating regions of the genome, both in the context of CpG and without CpG. In addition, differentially methylated regions were linked with endothelial and smooth muscle function [75]. 

Related research in pigs, which studied differential methylation in the ECs from an atherosensitive location (internal curvature of the aortic arch) and an athero-protected area (descending thoracic aorta), also showed a large number of hypermethylated sites that related to genes associated with transcription regulation, pattern-specific HOX loci, oxidative stress and an adaptive pathway to ER stress [76]. Moreover, hypermethylation of 5’UTR showed an inverse relationship with gene expression primarily at HOX loci. These observations correlate with changes in DNA methylation in aging or cancer, where global hypomethylation of megabase-sized genome blocks is a primary sign, even though aging is one of the risk factors for atherosclerosis [77].

A well-known molecular event in aging and senescence is the depression of repetitive elements with retrotransposon activation. Data on aging mouse cells and tissues indicate that these non-coding transcripts promoted from repeating elements are susceptible to reverse transcription and activate an interferon response that promotes systemic pro-inflammatory status during the process of aging [78]. It has been shown that the total coverage of 5hmC repetitive elements reduces during the development of the heart, but is elevated in the hypertrophied heart, especially with long intermittent nuclear elements. This was accompanied by a decrease in CG methylation and other repressive modifications of histones, which implies the subsequent activation of these regions in disease [79].

Most genome-wide methylation studies have been conducted using bead-based arrays, whole-genome bisulfite sequencing, or reduced-representation bisulfite sequencing after DNA bisulfite processing. Nevertheless, these methods do not reveal the difference between 5mC or 5hmC and can therefore complicate the mechanistic conclusions about gene regulation [80].

The oxidative bisulfite sequencing (oxBS-seq) new development method provides a chance to measure 5mC and 5hmC with single-nucleotide resolution in parallel. We are inclined to assume that these distinctive DNA modifications in cardiac aging and disease have not yet been fully studied and constitute a significant direction for future research [81].

## 9. Perspectives of Prevention

Those people who have an increased risk of developing CVD and/or those who have a family history of early manifestations of CVD are advised to screen and treat all common risk factors: hypertension, hyperlipidemia, impaired glucose tolerance or T2D (type 2 diabetes) and smoking, in accordance with current recommendations [82,83]. The results of studies aiming at decreasing known risks are summarized in Table 1. According to a study by INTERHEART, smoking is a risk factor for both dose-dependent coronary heart disease and vascular dysfunction in general, as well as the risk of high insulin resistance and T2D. New pharmacological treatments such as the use of a partial nicotine receptor agonist varenicline are able to help to get rid of nicotine addiction [84].

In addition, to protect the CV system it is important to improve the lifestyle in general, through a balanced diet, smoking cessation, and exercise. According to a study by INTERHEART, the consumption of fruits and vegetables, as well as moderate alcohol consumption, is important for maintaining a healthy CV system [85]. However, the most important appears to be physical activity. Thus, modern studies show that individuals with the highest physical activity rate demonstrate the lowest CVD risk [86,87]. Even small amounts of physical activity are linked to decreased CVD risk. The 2008 Physical Activity Guidelines Advisory Committee Report recommends 75 min/week of vigorous-intensity aerobic exercise and 150 min/week of moderate-intensity exercises to reduce CVD mortality. However, there is some apprehension that too much exercise training could produce deleterious cardiac effects, such as coronary calcification, myocardial infarction, and others [88,89]. In this regard, studies have shown that the benefits from physical exercise outweigh associated risks. 

Beneficial effects of physical exercises against vascular aging have been demonstrated [90,91,92]. Enhanced physical activity was shown to increase bioavailability of NO, reducing oxidative stress, and thus contributing to the anti-aging process.

These recommendations should be followed by many people with common risk factors below the limit levels; for example, with high blood pressure, cholesterol, or glucose, there is increased stiffness of the arteries, which is one of the signs of vascular aging. Alternative treatments for the prevention of manifestations of cardiovascular diseases based on atherosclerosis in patients at risk include the use of ACE inhibitors, as well as, according to recent large meta-analyses, statin therapy [93].

One interesting possibility is that in middle-aged men with short telomeres, treatment with pravastatin, may lead to a reduced risk of coronary heart disease, as described in the WOSCOP study. To date, it has not been fully proven whether angiotensin-2 (AT1) receptor blockers (ARBs) are able to provide the same, greater, or lesser vascular protection compared to a more studied class of ACE-inhibitors [94]. However, in a large-scale randomized ONTARGET trial involving more than 25,000 patients with hypertension and related risk factors, subjects received either the ACE suppressor ramipril or ARB telmisartan, or a combination of these two drugs to prevent fatal and non-fatal endpoints of cardiovascular disease [95,96]. 

**Table 1:** CVD risk factors and their management.

Study	Risk Factor	Drug	Effect	Reference
INTERHEART	Smoking	Varenicline	Nicotine addiction reduction	[84]
WOSCOP	Telomeres shortening	Pravastatin	CVD risk reduction	[94]
ONTARGET	Hypertension	Telmisartan and/or ramipril	CVD endpoints prevention	[95]

In 2006, a new treatment algorithm was presented by ADA and EASD. This particular treatment should serve as the basis for the treatment of diabetes and its hyperglycemia, including the first stage of lifestyle changes in combination with metformin [10]. As an additional further step, the use of insulin, sulfonylurea, or glitazones may be recommended, if appropriate. Another example of the new joint guideline ESC/EASD in Europe describes how to work for strict control of cardiovascular disease risk factors in all diabetic patients. In the case of effective use of these preventive measures, it would be possible to counteract the risk of early CV aging and slow down the development in these high-risk patients [97,98].

## 10. Conclusions

We are still far from a global understanding of the aging process. Yes, we understand which mechanisms are the main contributors, but the interaction between them still raises questions. Similarly, a causal relationship between aging and age-associated disease remains to be established, as there are several hypotheses on this score.

As for attempts to prevent or slow down aging, without a deep understanding of the complex interplay of multiple processes, they are somehow doomed to failure. Nevertheless, to prevent, if not aging in principle, then at least early aging is the cherished goal of many scientists, if not all of humanity. Some successful steps have been taken in this direction. It is important to note that these successful steps relate to the prevention of age-related diseases, such as type 2 diabetes, atherosclerosis, and others.

Summing up, we would like to emphasize once again that aging is a complex process, and only by systematizing all the accumulated information can one come to an understanding and modulation of aging.

## Data Availability

Not applicable.
